# Immunodominant HIV-1-specific HLA-B- and HLA-C-restricted CD8+ T cells do not differ in polyfunctionality

**DOI:** 10.1016/j.virol.2010.06.002

**Published:** 2010-09-30

**Authors:** Nompumelelo Mkhwanazi, Christina F. Thobakgale, Mary van der Stok, Shabashini Reddy, Zenele Mncube, Fundisiwe Chonco, Bruce D. Walker, Marcus Altfeld, Philip J.R. Goulder, Thumbi Ndung'u

**Affiliations:** a HIV Pathogenesis Programme, Doris Duke Medical Research Institute, University of KwaZulu-Natal, Durban, South Africa; b Ragon Institute of Massachusetts General Hospital, Massachusetts Institute of Technology and Harvard University, Charlestown, MA, 02129, USA; c Howard Hughes Medical Institute, Chevy Chase, MD, USA; d Department of Paediatrics, Peter Medawar Building for Pathogen Research, University of Oxford, Oxford OX13SY, UK

**Keywords:** HLA-B*57/5801, HLA-C, HIV-1 chronic infection, CD8+ T cells, Polyfunctionality

## Abstract

HIV-1 specific HLA-B-restricted CD8+ T cell responses differ from HLA-C-restricted responses in antiviral effectiveness. To investigate possible reasons for these differences, we characterized the frequency and polyfunctionality of immmunodominant HLA-B*57/B5801- and HLA-Cw*07-restricted CD8+ T cells occurring concurrently in nine study subjects assessing IFN-γ, TNF-α, IL-2, MIP-1β, and CD107a by flow cytometry and analyzed sequence variation in targeted epitopes. HLA-B*57/5801 and HLA-Cw*07 restricted CD8+ T cells did not differ significantly in polyfunctionality (p = 0.84). Possession of three or more functions correlated positively with CD4+ T cell counts (r = 0.85; p = 0.006) and monofunctional CD8+ T cells inversely correlated with CD4 cell counts (r = −0.79; p = 0.05). There were no differences in polyfunctionality of CD8+ T cells specific to wildtype versus mutated epitopes. These results suggest that loss of polyfunctionality and increase in monofunctional HIV-1-specific CD8+ T cells are associated with disease progression independent of restricting HLA allele. Furthermore, sequence variation does not appear to significantly impact CD8+ T cell polyfunctionality in chronic HIV-1 infection.

## Introduction

HIV-specific CD8+ T cells play a vital role in the control of HIV replication and disease progression (Altfeld et al., 2006; Borrow et al., 1994; Koup et al., 1994; Schmitz et al., 1999). However, differences exist in their antiviral effectiveness based on their HLA restriction, epitope specificity, functional epitope avidity and targeted viral protein (Bennett et al., 2007; Bihl et al., 2006; Kiepiela et al., 2007). In particular, major histocompatibility complex (MHC) class I molecules have been shown to differ in their ability to mediate the control of HIV and SIV replication in humans and non-human primates respectively (Goulder and Watkins, 2008; Kiepiela et al., 2004). For example, virus-specific Gag CD8+ T cell responses restricted by HLA-B*57, HLA-B*5801 and HLA-B*27 are associated with low viral loads or slow disease progression in HIV infection (Kiepiela et al., 2007; Klein et al., 1998; Migueles and Connors, 2001; Novitsky et al., 2003) while Mamu-A*01 and Mamu-B*17 restricted responses are associated with control in SIV infection of rhesus macaques (Chung et al., 2007; Loffredo et al., 2007; Maness et al., 2008; Migueles et al., 2003).

In contrast to the beneficial outcomes associated with the protective MHC allele-restricted Gag CD8+ T cell responses, Gag HLA-C-restricted CD8+ T cell responses were found to be associated with high viral loads (Kiepiela et al., 2007). Paradoxically, although HLA-C-restricted CD8+ T lymphocytes appear to contribute little or even negatively to viral control *in vivo*, HLA-C is not down-regulated by HIV-1 Nef from the surface of infected cells to the same extent that HLA-A and HLA-B molecules are (Cohen et al., 1999; Collins et al., 1998). Overall, the mechanisms underlying control of HIV by protective alleles such as HLA-B*57/5801, or the lack of control by HLA-C alleles remain unclear and this limited understanding has important implications for rational vaccine design.

HIV-specific CD8+ T cells may also display different differentiation status and activation profiles (Appay and Sauce, 2008; Papagno et al., 2004). It has been suggest that these phenotypic differences are associated with divergent functional antiviral capacities of virus-specific T cells (Almeida et al., 2009). Some studies have suggested that polyfunctional CD8+ T cells, able to secrete up to five different effector functions (IFN-γ, IL-2, TNF-α, MIP-1β and CD107a), have better antiviral activity (Betts et al., 2006; Daucher et al., 2008; Precopio et al., 2007). CD8+ T cell responses restricted by HLA-B*27 and HLA-B*57 alleles were reported to be polyfunctional when compared to CD8+ T cell responses restricted by HLA-A alleles within the same patients (Harari et al., 2007). However, recent data have suggested that the functional profile of CD8+ T cells is largely a consequence of the duration and level of antigen load, with prolonged continuous exposure to high levels of antigen resulting in exhausted CD8+ T cells characterized by a monofunctional effector profile (Rehr et al., 2008; Streeck et al., 2008a).

Here, we studied the polyfunctionality profiles of immunodominant HLA-B and HLA-C-restricted CD8+ T cells in a cohort of HIV-1 clade C chronically infected individuals displaying both responses. This provided the unique opportunity to examine HLA-B and C restricted responses in the context of matched viral loads and CD4 cell counts. We focused on the immunodominant HLA-B*57/*5801 epitopes in Gag p24 and the immunodominant HLA-Cw*07 restricted epitope KY11 in Nef, in persons possessing both responses. We hypothesized that HLA-B CD8+ T cells will display a more polyfunctional phenotype compared to HLA-C-restricted CD8+ T cells. We also aimed to determine whether sequence variation within epitopes presented by these two alleles has a bearing on the magnitude and polyfunctionality of epitope-specific CD8+ T cell responses. The relationship between the frequency of polyfunctional HIV-1-specific CD8+ T cells and CD4+ T cell counts and viral loads was also investigated.

## Results

### Characteristics of study subjects

The study subjects were seven females and two males, with a median age of 40 (range 27–58) years, all coexpressing HLA-B*57/5801 and Cw*07. The subjects were selected on the basis of possession of concurrent dominant HLA-B*57/5801 and HLA-Cw*07 restricted HIV-1 specific CD8+ T cell responses ≥500 SFC/10^6^ on IFN-γ ELISPOT. The median plasma viral load was 11,500 (range 2530–750,000) RNA copies/ml and the median CD4 count was 271 (range 202–411) cells/μl. The subject gender, age, CD4 count, viral load, HLA type and epitopes examined for each subject are shown in Table 1. These subjects were selected from the Sinikithemba cohort, which compromised of 451 HIV-1 infected individuals whose time of infection was unknown. In the cohort, 37 of 451 (8.2%) participants coexpressed HLA-B*57/5801 and HLA-Cw*07, 51 (11.3%) expressed HLA-B*57/5801 without HLA-Cw*07 and 81 (18%) expressed HLA-Cw*07 without HLA-B*57/5801. The median age CD4 cell count and viral load for the 37 subjects who coexpressed HLA-B*57/5801 and HLA-Cw*07 was 37 years, 490 cells/μl and 6700 copies/ml respectively. Of these 37 individuals only the nine further studied here had concurrent immunodominant HLA-B*57/5801- and HLA-Cw*07-restricted IFN-γ ELISPOT responses defined as ≥ 500 SFC/10^6^ PBMCs.

### Magnitude and breadth of HLA-B*57/5801 and HLA-C restricted responses by the IFN-γ ELISPOT assay

**Fig. 1:**
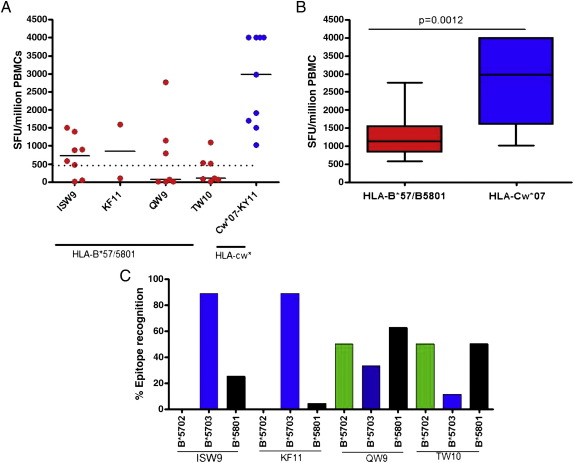
Measurement of HLA-B*57/5801 and HLA-Cw*07-restricted T cell responses by Interferon-gamma ELISPOT assay. (A) Hierarchy of dominant epitopes presented by the study subjects expressing HLA-B*57/5801 and HLA-Cw*07 alleles (n = 9). Dotted line indicates responses above 500 SFCs from ELISPOT assay that were further tested in subsequent multicolor assays. (B) Combined total magnitude of T cell responses presented by both HLA-B*57/B5801 and HLA-Cw*07 restricted epitopes in the study individuals (n = 9). (C) Percentage of epitope recognition (>100 SFCs/10^6^ ELISPOT responses) of the dominant epitopes presented by different HLA-B57/5801 alleles (B*5701, B*5703 and B*5801) in all chronically infected subjects coexpressing HLA-Cw*07 (n = 37).

All subjects included in this study made HIV-specific CD8+ T cell responses to known HLA-B*57/5801 and HLA-Cw*07 epitopes as determined by IFN-γ ELISPOT (Fig 1A). Only a few individuals within the Sinikithemba study cohort had responses ≥ 500/10^6^ SFCs for both HLA-B*57/5801 and HLA-Cw*07; these high magnitude responses were examined further in subsequent assays. The immunodominant HLA-B*57/5801-restricted responses were to the following four epitopes: TSTLQEQIAW (TW10), ISPRTLNAW (ISW9), QATQDVKNW (QW10) and KAFSPEVIPMF (KF11). In contrast, only one HLA-C response was targeted by the study subjects: KRQEILDLWVY (KY11) restricted by HLA-Cw*07 (Fig 1A; Suppl. Table 1). The overall magnitude of the responses targeted by HLA-Cw*07 restricted epitopes was significantly higher than the magnitude of HLA-B*57/5801 restricted responses (p = 0.0012, Mann–Whitney test; Fig 1B).

The nine study patients were representative of an additional 28 individuals (total n = 37) (data not shown) with HLA-B*57/5801-restricted responses in terms of breadth of positive IFN-γ epitope-specific ELISPOT responses detected. ISPRTLNAW (ISW9) and KAFSPEVIPMF (KF11) were dominantly presented by HLA-B*5703; whereas the presentation of QW9 and TW10 was variable between B*5702 and B*5801 and were least presented by HLA-B*5703 (Fig. 1C). Notably, none of the B*5702 subjects presented ISW9 and KF11.

### Functionality profiles of HLA-B*57/5801 and HLA-C restricted HIV-1 specific CD8+ T cell epitopes

Previous studies of long term non-progressors have shown that HIV-1-specific CD8+ T cells restricted by protective alleles such as HLA-B*57 and HLA-B*27, may be more polyfunctional than CD8+ T cells restricted by other HLAs (Champagne et al., 2001; Harari et al., 2007; Zimmerli et al., 2005). To determine the functionality of HIV-specific CD8+ T cells restricted by alleles with different disease outcomes (HLA-B*57/5801 and HLA-Cw*07); we assessed the polyfunctionality of these responses in those who possessed them concurrently.

**Fig. 2:**
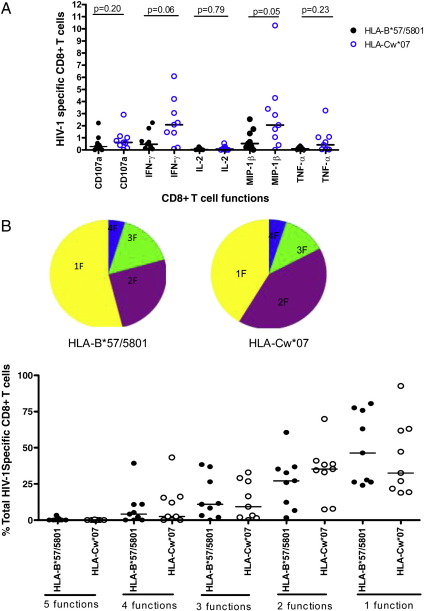
Assessment of HIV-1 specific CD8+ T cell polyfunctionality by multicolor staining for HLA-B*57/5801 and HLA-Cw*07-restricted epitopes. (A) Magnitude of HLA-B*57/5801 and HLA-C restricted epitope responses using multicolor staining, single CD8+ T cell function responses are shown after background subtraction. (B) Comparison of the contribution of individual functions between HLA-B*57/B5801 (○) and HLA-C (●) restricted epitopes in the study subjects (*n* = 9). The fractions of the response patterns are grouped and color-coded by the number of functions and summarized in pie chart form where each slice of the pie represents the fraction of the total epitope-specific response that consist of CD8+ T cells with the respective number of functions.

HIV-specific CD8+ T cells polyfunctionality was evaluated using multicolor flow cytometry by simultaneous measurement of five functions: IFN-γ, TNF-α, IL-2, MIP-1β, and CD107a as previously described in other studies (Betts et al., 2006; De Rosa et al., 2004; Streeck et al., 2008a). On single function gating (Supp. Fig. 1), IFN-γ expression was lower on HLA-B*57/5801 than on HLA-Cw*07 restricted HIV-1 specific CD8+ T cell epitopes (p = 0.06; Mann–Whitney test) (Fig. 2A), consistent with ELISPOT data. A similar trend was observed for the other individual functions although the magnitude of other responses was lower than for IFN-γ.

The vast majority of both HLA-B and HLA-C restricted CD8+ T cells (75%) in this study cohort were monofunctional (Fig. 2B). The most frequently expressed function was MIP-1β at 40% for HLA-B*57/5801 and 30% for HLA-Cw*07, with CD107a at 30% for HLA-B*57/5801 restricted CD8+ T cells and 28% for HLA-C; and IFN-γ at 29 % for HLA-B*57/5801 versus 30% for HLA-C. Monofunctional IL-2 or TNF-α producing HIV-specific CD8+ T cells were very infrequent in this cohort of chronically infected patients and only a small proportion of HIV-specific CD8+ T cells expressed TNF-α responses confirming previous studies that this effector function is lost early in infection (Lichterfeld et al., 2004; Wherry et al., 2003).

To further investigate potential differences in functional profiles upon stimulation with both HLA-B*57/5801- and HLA-Cw*-restricted peptides on all patients, we compared the frequencies of the different HLA-restricted CD8+ T cells expressing different functions detected in these individuals. The fraction of each function was determined as a percentage of the total CD8+ T cell response as previously described (Streeck et al., 2008b). No significant differences were observed between HLA-B*57/5801- and HLA-Cw*-restricted responses with regard to five (p = 0.64; Mann–Whitney test), four (p = 0.89), three (p = 0.87), two (p = 0.22) and one function (p = 0.37) (Fig. 2B). Taken together, these data suggest the differences in disease outcome observed for HLA-B*57/5801 and HLA-Cw* cannot be explained by the polyfunctionality of the HIV-specific CD8+ T cell responses during chronic infection. In addition, polyfunctionality of CD8+ T cell responses may not depend on the restricting HLA allele during the chronic phase of infection.

### Relationship between the polyfunctionality of HLA-restricted HIV-1 specific CD8+ T cells, CD4 counts and viral loads

**Fig. 3:**
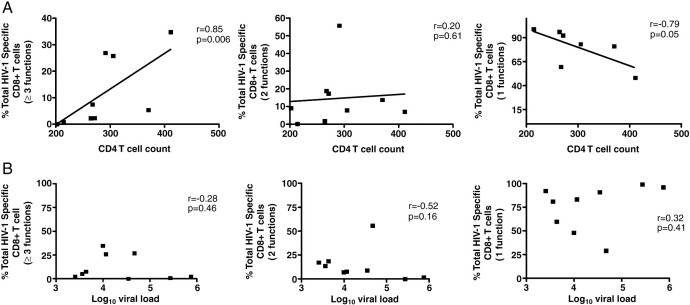
Relationship between the fractions of monofunctional, bi-functional and polyfunctional HIV-1 specific CD8+ T cell responses and the (A) CD4+ T cell counts and (B) viral loads.

Previous studies have suggested that HLA-B-restricted HIV-1-specific CD8+ T cells are more polyfunctional than HLA-A and HLA-C (Harari et al., 2007; Zimmerli et al., 2005) and that the proportion of the HIV-1-specific CD8+ T cell with the highest functionality inversely correlate with viral loads in non-progressive(Betts et al., 2006). The proportion of HIV-1-specific CD8+ T cell responses were plotted against CD4 counts and viral load, for all the functions (Figs. 3A and B). Since no differences were noted in polyfunctionality profiles of HLA-B*57/5801 and Cw*07-restricted epitopes, the total percentages across different (1–5) functions were added together to determine whether or not there was a relationship between the polyfunctional CD8+ T cells, viral load and CD4 counts. We noted a significant positive correlation between 3 or more functions and CD4 counts (p = 0.006; r = 0.85, Spearman test) (Fig. 3A). In contrast, there was a negative correlation between monofunctional cells and CD4 counts (p = 0.05; r = −0.79). However, no correlation was noted between the polyfunctional or monofunctional CD8+ T cells and viral loads (Fig 3B).

### Sequence variation within HLA-B*57/5801 and HLA-C restricted epitopes and its impact on CD8+ T cell polyfunctionality

Sequence variation and the accumulation of mutations over course of infection are known to affect epitope recognition, specificity of the T cell receptor binding and recognition by HLA (Goulder et al., 1997; Nixon et al., 1988).Next, we evaluated whether the low magnitude of responses noted in HLA-B*57/5801 compared to high HLA-Cw*07-restricted CD8+ T cell responses was due to the sequence variation in the CD8+ T cell epitopes. *Gag* and *nef* genes were sequenced in all nine patients as the epitopes studied here were located in these viral proteins. HLA-B*57/5801-restricted HIV-1-specific CD8+ T cell epitopes had more sequence variation (6/9) when compared to HLA-Cw*07-restricted epitopes (1/9), (p = 0.05; Fisher's exact test), despite the fact that the HLA-C-restricted response examined is in the highly variable Nef protein. The most frequent sequence changes were noted in the TSTLQEQIAW (TW10) and ISPRTLNAW (ISW9) Gag epitopes restricted by HLA-B*57/5801, and a single sequence variation was noted in the Nef Cw*07-restricted epitope KRQEILDLWVY (KY11) restricted by HLA-Cw*07 (Table 2). These data suggest that HLA-B*57/5801-restricted epitopes have high sequence variation compared to HLA-Cw*07-restricted epitopes and this may in part account for the low magnitude of HLA-B*57/5801- compared to HLA-Cw*07-restricted responses.

**Fig. 4:**
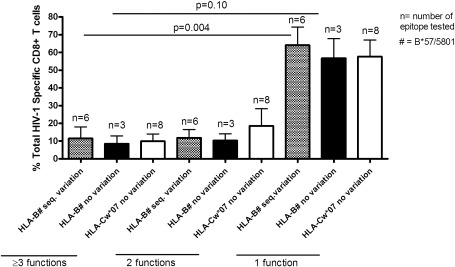
Evaluation of the relationship between epitope sequence variation and polyfunctionality of HIV-specific CD8+ T cell responses. HLA- B*57/5801 and HLA-Cw*07 restricted epitope responses with and without sequence variation were plotted against the percentage of total HIV-1 specific CD8+ T cells with ≥ 3 functions, bi-functional and monofunctional CD8+ T cell responses generated from the study individuals (n = 9).

We also investigated whether or not sequence variation impacted on CD8+ T cell polyfunctionality. CD8+ T cell polyfunctionality was assessed by comparing HLA-B and HLA-C-restricted HIV-1-specific CD8+ T cell epitopes with or without the sequence variation against the percentage of the total HIV-1-specific CD8+ T cell response (Fig. 4). No differences were noted between epitopes with or without sequence variation with regard to 3 or more functions, 2 functions and monofunctional T cells. However, there was a high frequency of monofunctional HIV-1-specific CD8+ T cells when compared to other functions e.g. monofunctional HLA-B*57/5801 epitopes with or without sequence variation were higher than HLA-B*57/5801 epitopes with or without sequence variation expressing 3 or more functions (p = 0.004, p = 0.10 respectively, Mann–Whitney test). Thus, whereas the decrease in the frequency of the HLA-B*57/5801-restricted responses compared to HLA-Cw*07-restricted CD8+ T cell responses may have been due to sequence variation, these data imply that sequence variation alone may not affect the polyfunctionality of HLA-B*57/5801 or HLA-Cw*07 restricted HIV-1-specific CD8+ cells.

## Discussion

The mechanisms underlying better control of HIV-1 by certain HLA alleles are not well understood. Understanding these mechanisms could facilitate the rational design of an effective HIV-1 vaccine. We therefore here investigated the functional characteristics of CD8+ T cells responses restricted by either HLA-B*57/5801 or HLA-Cw*07 alleles within the same individuals in order to better understand the differences in disease outcome mediated by these two alleles.

We show that IFN-γ producing CD8+ T cell responses restricted by HLA-Cw*07 were significantly higher than responses mediated by HLA-B*57/5801, both on ELISPOT and intracellular cytokine staining (Figs. 1B and 2A). As shown in Fig 1C, the targeted responses in the nine study subjects were representative of other HLA-B*57/5801-possesing persons in the larger cohort. However, the nine study participants were in a relatively advanced stage of infection, as evidenced by the low median CD4+ T cell count of 288 cells/μl, and it is possible that this disease progression status may have influenced the magnitude of HLA-B*57/5801-restricted responses relative to HLA-C responses. On the other hand, the more obvious explanation of the high magnitude of HLA-C-restricted responses is the higher conservation of these epitopes compared to the escaped HLA-B*57/5801-restricted epitopes (Table 2). We also noted that in the Sinikithemba cohort from which the nine participants studied here were selected, there was significantly higher B*57/5801-restricted responses in individuals coexpressing HLA-Cw*07 compared to those HLA-B*57/5801-positive subjects who do not express HLA-Cw*07 (data not shown). Also, Cw*07 restricted responses were significantly higher in those coexpressing B*57/5801 compared to those not expressing B*57/5801. These data suggest that there may be some interactions between these alleles that influence the magnitude of immune responses. Further studies will be needed to address whether there are differences in polyfunctionality of T cells restricted by these alleles in individuals coexpressing them versus those expressing one allele without the other.

We hypothesized here that protective HLA-B restricted CD8+ T cells have a more polyfunctional profile compared to the non-protective HLA-C-restricted responses as has been described in some studies (Betts et al., 2006; De Rosa et al., 2004). In contrast to these earlier studies, we found no significant differences in polyfunctional CD8+ T cell responses mediated by the two alleles within patients with both responses (Fig. 2B). Whereas in the other studies differences were analyzed between patients, here we studied responses occurring concurrently within a patient thus eliminating confounding by disease status, environmental and genetic factors. We observed a positive correlation between polyfunctionality and CD4+ T cell count, but no correlation with viral load suggesting that disease progression but not viral antigen load *per se* is associated with loss of CD8+ T cell polyfunctionality (Fig. 3A). The lack of correlation between the polyfunctionality of HIV-1 specific CD8+ T cells and viral load is in contrast to results from earlier studies (Betts et al., 2006; Daucher et al., 2008) and it is possible that the small sample size in our study and the relatively advanced phase of infection for all the study subjects reducing the power to detect differences. We also observed an inverse correlation between the proportion of monofunctional CD8+ T cells and CD4+ T cell counts, which suggests that immune dysfunction as seen in late chronic HIV-1 infection is characterized by increasing proportion of exhausted monofunctional cells.

It was recently observed in a longitudinal study of recent HIV-1 infection that CD8+ T cells directed against conserved epitopes lost their polyfunctionality whereas escaping-epitope targeting CTLs appeared to maintain their original polyfunctional profile (Streeck et al., 2008a). We investigated here whether there were sequence variation differences in HLA-B*57/5801 versus HLA-Cw*07-restricted epitopes and whether such differences resulted in divergent CD8+ T cell polyfunctionality profiles HLA-B*57/5801-restricted epitopes were more variable, perhaps suggesting increased immune selection pressure on these epitopes, however, there were no differences observed in polyfunctionality between these and immunodominant HLA-Cw*07 restricted CD8+ T cells. Although limited by small sample size, these data imply that sequence variation in targeted epitopes may have no impact on the level of polyfunctionality of CD8+ T cells during chronic HIV-1 infection, although as shown previously, sequence variation within a targeted epitope can impact on polyfunctionality over time (Streeck et al., 2008b). We emphasize that our results should not be interpreted to be contradicting those of Streeck and colleagues given the differences in study design; here we used a cross-sectional design to analyze polyfunctionality differences in CD8+ T cells to conserved versus variable epitopes compared to longitudinal follow-up of escaping or non-escaping epitopes in the earlier study. Furthermore, in the current study we cannot tell how long the sequences noted for the epitopes had existed in the patients without undergoing changes, a factor that may affect polyfunctionality. In addition, the population sequencing strategy we employed may limit the detection of all variants present in the study subjects. It is also possible that not all escape mutants have the same impact, in some cases variants continue to be partially recognized and a CD8+ T cell response is maintained whilst in other cases, for example where HLA-binding is abrogated, the CD8+ T cell response falls to very low levels. We speculate that the result of these two extremes on polyfunctionality would differ considerably, such that partial reduction in epitope recognition would decrease or otherwise change CD8+ T cell polyfunctionality minimally, whereas more complete loss of epitope recognition would significantly increase CD8+ T cell polyfunctionality.

The strength of this study is that CD8+ T cells were analyzed concurrently within study subjects with both responses of interest, thus eliminating across subject confounders. However, limitations include a small sample size and relatively advanced phase of infection of study subjects. We also did not measure CD8+ T cell antiviral function directly and are thus unable to conclude whether polyfunctionality had any association with antiviral functional capacity.

In summary, we found that in late chronic HIV-1 infection, immunodominant HLA-B*57/5801 restricted IFN-γ CD8+ T cell responses were of lower magnitude compared to immunodominant HLA-Cw*07-restricted responses in patients with both responses possibly due to differences in sequence variation in targeted epitopes. We did not find evidence of polyfunctionality differences between HLA-B*57/5801 versus HLA-C-restricted CD8+ T cells. Polyfunctionality of CD8+ T cells correlated positively with CD4 T cell counts, suggesting that either polyfunctionality is lost as disease progresses or that loss of polyfunctionality leads to disease progression. There was no impact of sequence variation within targeted epitopes on the polyfunctionality of restricted CD8+ T cells. Larger longitudinal studies are needed to better elucidate the mechanisms that underlie protective versus non-protective HLA-mediated CD8+ T cell responses.

## Materials and methods

### Study subjects

Nine HIV-1 infected patients were selected for this study from the Sinikithemba cohort, a prospective natural history study of HIV-1 chronically infected, antiretroviral naïve individuals, established in 2003 at McCord Hospital, Durban, South Africa (Kiepiela et al., 2004; Kiepiela et al., 2007). In this study population, Cw*0701 is in linkage disequilibrium with B*5801 (p = 0.0239) and B*5703 (p = 0.0097) and B*5702 (p = 0.0034) as determined by the HLA linkage disequilibrium tool on the Los Alamos HIV database (http://www.hiv.lanl.gov/content/immunology/hla/hla_linkage.html). In B*5702/5703-positive subjects, 64% coexpress Cw*07, and in B*5702/5703-positive subjects who do not express Cw*0701, 97% coexpress Cw*1801. In B*5801-positive subjects, 65% coexpress Cw*07 and in 88% of the remainder either Cw*0302 or Cw*0602 are coexpressed with B*5801.

The nine subjects studied here were selected based on the possession of concurrent immunodominant HLA-B*57/5801- and HLA-Cw*07-restricted CD8+ T cell responses detected by gamma interferon (IFN-γ) ELISPOT, defined as a minimum magnitude of 500 spot forming cells per million (SFC/million) PBMC for each of these responses.

### Viral load and CD4 counts measurement

Viral loads (VL) were determined from plasma using Roche Amplicor (version 1.5) and CD4+ T cell counts were enumerated from fresh blood by Tru-Count technology using a four-color FacsCalibur flow cytometer (Becton Dickinson) as previously described (Ngumbela et al., 2008; Thobakgale et al., 2007).

### HLA typing

DNA for HLA typing was isolated using Puregene DNA isolation kit for blood (Gentra systems, Minneapolis, MN) according to the manufacturer's instructions. HLA class I typing was done by DNA PCR using sequence-specific primers as described before (Kiepiela et al., 2004; Thobakgale et al., 2009).

### Synthetic HIV-1 peptides and Interferon-γ ELISPOT assay

A panel of 410 overlapping peptides (18mers with 10–12 amino acid overlap) spanning the entire HIV-1 clade C consensus sequence were synthesized and used in matrix screening assays as previously described (Goulder et al., 2001; Kiepiela et al., 2004). Previously defined optimal peptides were similarly synthesized. *Ex vivo* measurement of T cells for IFN-γ production was undertaken by the ELISPOT assay as previously reported (Kiepiela et al., 2004; Ngumbela et al., 2008; Thobakgale et al., 2009). The antigen-specific T cell responses were considered positive if they were > 100 SFC above the unstimulated negative control wells.

### Polyfunctionality analysis by multicolor flow cytometry

*Ex vivo* measurement of CD8+ T cells for expression of IFN-γ, IL-2, MIP-1β, TNF-α and CD107a was assessed by multicolor flow analysis as previously described (Streeck et al., 2008a). In brief, freshly thawed cryopreserved PBMCs were resuspended to 1–2 × 10^6^ cells/ml in R10 media (RPMI 1640 supplemented with 10% heat-inactivated FCS, 100 U/ml penicillin, 1.7 mM sodium glutamate, 5.5 ml HEPES buffer) and rested for 2 h at 37 °C and 5% CO_2_. One million cells/ml were stimulated with optimal HLA-B*57/5801 or HLA-Cw*-restricted peptides that represented immunodominant (≥ 500 SFC on ELISPOT) responses in the study subjects (Fig. 1A) in the presence of anti-CD28 and anti-CD49 co-stimulatory antibodies. A negative control with PBMCs alone and a positive control containing PBMCs stimulated with *Staphylococcus* enterotoxin B (SEB) were included in the assays. Anti-CD107a-PE-Cy5 (BD Biosciences) antibody was added and incubated for 30 min at 37 °C, 5% CO_2_, followed by addition of Brefeldin A (10 μg/ml, Sigma-Aldrich, St Louis, MO) and Monensin (2.5 μg/ml, Sigma-Aldrich) and incubated at 37 °C, 5% CO_2_ for total of 6 h. The cells were then washed with PBS (2% FCS), stained to differentiate between live/dead cells (violet viability dye, Invitrogen) and incubated for 30 min at 4 °C. Cells were then washed and stained with the following surface antibodies: anti-CD3-PE Cy5.5 (Caltag), CD4-APC, CD8-APC Cy7 (both from BD Biosciences) and incubated for 20 min in the dark at room temperature. Cells were again washed and fixed in 1% paraformaldehyde (Fix Perm A, Caltag) for 20 min in the dark at room temperature and were then permeabilized (Fix Perm B, Caltag) and stained intracellularly with the following antibodies: anti-IFN-γ PE Cy7, anti-TNF-α Alexa 700, anti-MIP-1β PE, anti-IL-2 FITC (all from BD Biosciences) before incubation for 20 min in the dark at room temperature. The cells were washed twice with PBS and resuspended in 200 μl of PBS before acquisition on LSRII flow cytometer (BD Bioscience).

### Sample acquisition and analysis

Between 200,000 and 1,000,000 events were collected per sample. Analysis was performed using DIVA and FlowJo 8.3.3 software (TreeStar, Ashland, OR). Initial gating was on the lymphocytes population, then forward scatter height (FSC-H) versus forward scatter area (FSC-A) to remove doublets (Supplementary Fig. 1). Subsequently, live CD3+ T cells gating, followed by identification of CD8+ T cells was done; then individual gates (set based on the negative control), for respective functions were made to identify positive responses. Boolean gating was performed to create a full array of possible combination of up to 32 response patterns. Positive responses were reported after background correction and the percentage of epitope-specific CD8+ T cell responses had to be at least two times higher than background for each tested marker. Analysis of multifunctional data was performed by PESTLE (version 1.6.2) and SPICE 5.0 (Mario Roederer, ImmunoTechnology Section, Vaccine Research Center, NIH, Bethesda, MD).

### Sequencing of *Gag* and *Nef* genes

HIV-1 RNA was extracted from 500 μl of plasma (VL < 5000 HIV-1 RNA copies/ml) or 140 μl plasma (VL > 5000 RNA copies) using QIAmp viral RNA extraction kit (Qiagen). RNA was reversed transcribed using one-step RT-PCR kit (Invitrogen) and gene specific primers under the following conditions: reverse transcription at 55 °C for 30 min, followed by amplification at 94 °C for 2 min, and 35 cycles of 94 °C for 15 s, 55 °C for 30 s, 68 °C for 2 min; and final extension at 68 °C for 5 min. *Gag* PCR primers were 5′-CTAGCAGTGGCGCCCGAACA-3′ and 5′GCAGTCTTTCATTTGGTGTCCTCC-3′. The same primers were used in the second round PCR reaction using the following conditions: 94 °C for 2 min, followed by 35 cycles of 94 °C for 15 s, 58 °C for 30 s, 72 °C for 1:30 min, with a final extension at 72 °C for 7 min. Nef PCR primers were 5′-TTCAGCTACCACCGATTGAGA-3′ and 5′-TGAGGGTTGGCCACTCC-3′. The PCR conditions were similar to those for *gag* except for the second round PCR reaction, where the annealing temperature was set at 56 °C for 30 s. Purified (Qiagen) PCR products were sequenced using BigDye v3.1 Terminator sequencing kit (Applied Biosystems, Foster city, CA, USA) on a XL-3100 automatic DNA sequencer (Applied Biosystems). Nucleotide sequences were analyzed using Sequencer 4.8 software (GeneCodes Corporation, Ann Arbor, MI). Alignments of reference and newly generated *gag* and *nef* sequences were performed using ClustalX and edited by Bioedit Sequence Alignment editor.

### Statistical analysis

Mann–Whitney U test was used to compare the median magnitude of HLA-B versus HLA-C responses. The same test was used to compare individual effector functions or polyfunctional responses between HLA-B*57/5801 and HLA-C responses in individuals. The Spearman rank correlation test was used to correlate individual and polyfunctional responses with viral loads and CD4 counts. Fisher's exact test was used to compare proportions of HLA-B and HLA-C epitopes with sequence variation.

## Figures and Tables

**Table 1:** Characteristics of study subjects.

Patient ID	Sex	Age (years)	CD4 count (cell/ml)	Viral load (copies/ml)	HLA type	HLA-B*57/5801 epitopes	HLA-C epitopes
SK 009	Male	32	291	47,000	A*2301/74 B*1503/5702 Cw*0202/0701	TSTLQEQIAW (p24)	KRQEILDLWVY(Nef)
SK 215	Female	35	202	34,800	A*6802/74 B*0702/5703 Cw*07/07	ISPRTLNAW (p24)	KRQEILDLWVY(Nef)
SK 236	Female	37	411	9900	A*02/3002 B*0801/5801 Cw*07/07	ISPRTLNAW (p24)	KRQEILDLWVY(Nef)
SK 251	Female	58	271	2530	A*02/3001 B*4201/5801 Cw*07/1701	QATQDVKNW (p24)	KRQEILDLWVY(Nef)
SK 318	Female	27	370	3600	A*33/74B*0702/5703 Cw*07/07	KAFSPEVIPMF (p24)	KRQEILDLWVY(Nef)
SK 358	Female	42	264	750,000	A*0202/2301 B*08/5701 Cw*07/07	ISPRTLNAW (p24)	KRQEILDLWVY(Nef)
SK 364	Female	38	305	11,500	A*02/3001 B*4201/5801 Cw*07/1701	QATQDVKNW (p24)	KRQEILDLWVY(Nef)
SK 379	Male	44	267	4310	A*0205/0208 B*0702/5801 Cw*07/07	TSTLQEQIAW (p24)	KRQEILDLWVY(Nef)
SK 428	Female	45	214	272,000	A*0205/0208 B*1401/5801 Cw*07/08	TSTLQEQIAW (p24)	KRQEILDLWVY(Nef)
Median		38	271	11,500			

**Table 2:**
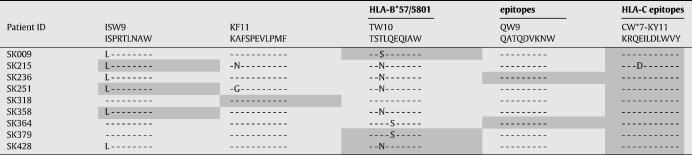
Sequence variation in CD8+T cell epitopes presented by HLA-B*57/B5801 and HLA-Cw*07 alleles.

Highlighted-epitopes tested in ICS. Unhighlighted-epitopes not tested in ICS.

## Appendix A Supplementary data

Supplementary Fig. 1 Representative gating strategy for the identification of polyfunctional CD8+ T cells after stimulation with either HLA-B*57/58 or HLA-Cw* 07 peptides are shown.

Supplementary Table 1 ELISPOT responses of HLA-B*57/5801- and HLA-C-Restricted epitopes for each patient
