# Supplementary figures and images for: Immunodominant HIV-1-specific HLA-B- and HLA-C-restricted CD8+ T cells do not differ in polyfunctionality
Source: Virology. 2010 Sep 30;405(2-3):483–91. doi: 10.1016/j.virol.2010.06.002 (PMC2954365; doi:10.1016/j.virol.2010.06.002)

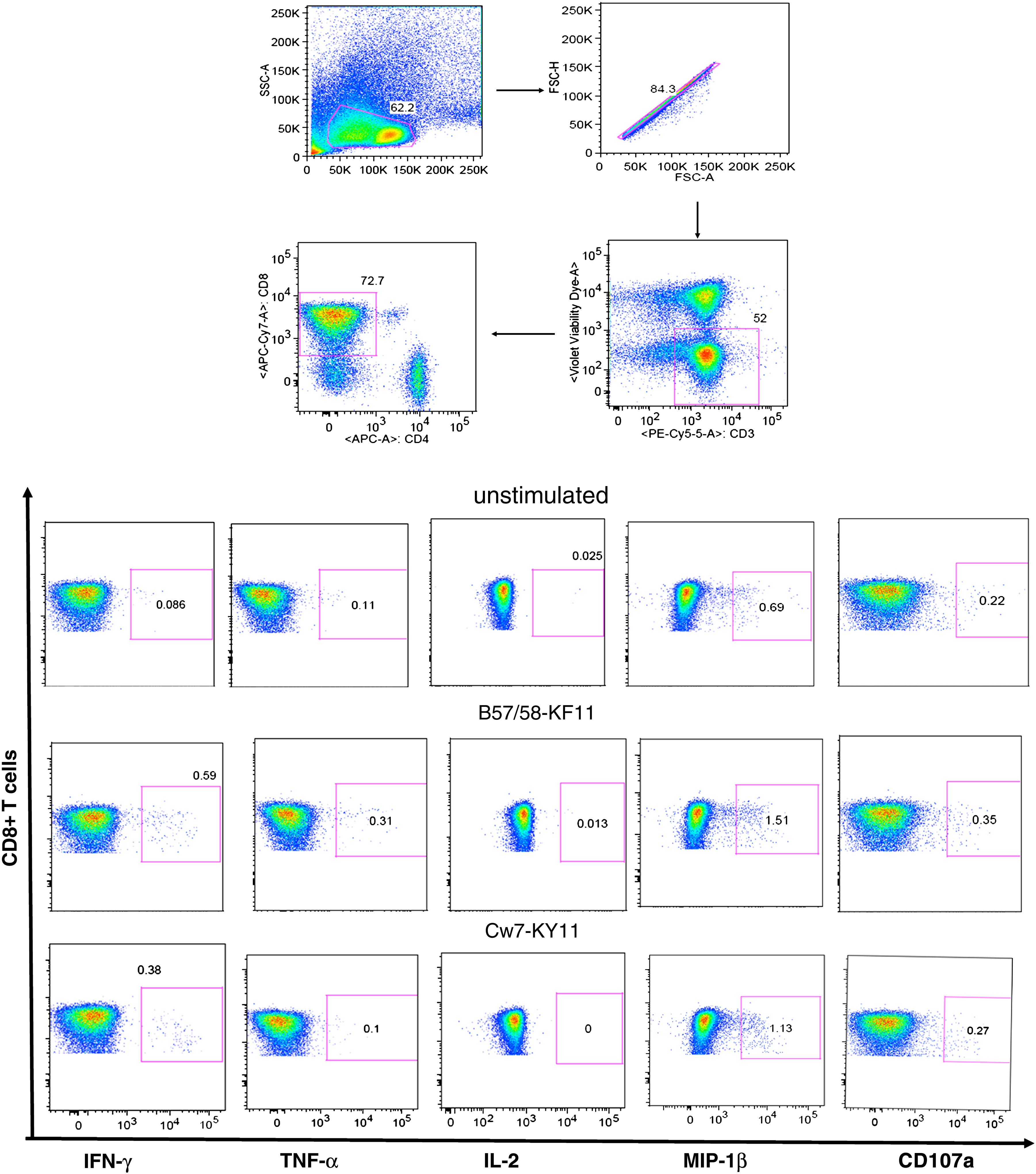

Supplement: Supplementary file 1 [file gr5.jpg]
