# Supplementary material for: Immunodominant HIV-1-specific HLA-B- and HLA-C-restricted CD8+ T cells do not differ in polyfunctionality
Source: Virology. 2010 Sep 30;405(2-3):483–91. doi: 10.1016/j.virol.2010.06.002 (PMC2954365; doi:10.1016/j.virol.2010.06.002)
Supplement: Supplementary Fig. 1 — Representative gating strategy for the identification of polyfunctional CD8+ T cells after stimulation with either HLA-B*57/58 or HLA-Cw* 07 peptides are shown. [file mmc1.pdf]

Supplementary Table 1: ELISPOT responses of HLA-B*57/5801- and HLA-C- Restricted epitopes for each patient

Patient ID	HLA-B*57/5801 epitopes	Magnitude of responses SFU/million	HLA-Cw *0701 epitopes	Magnitude of responses SFU/million
SK 009	TSTLQEQIAW (p24)	500	KRQEILDLWVY (Nef)	4000
SK 215	ISPRTLNAW (p24)	1400	KRQEILDLWVY (Nef)	4000
SK 236	ISPRTLNAW (p24)	900	KRQEILDLWVY (Nef)	2980
SK 251	QATQDVKNW (p24)	800	KRQEILDLWVY (Nef)	1500
SK 318	KAFSPEVIPMF (p24)	1600	KRQEILDLWVY (Nef)	1920
SK 358	ISPRTLNAW (p24)	1500	KRQEILDLWVY (Nef)	1700
SK 364	QATQDVKNW (p24)	1140	KRQEILDLWVY (Nef)	1020
SK 379	TSTLQEQIAW (p24)	520	KRQEILDLWVY (Nef)	4000
SK 428	TSTLQEQIAW (p24)	1100	KRQEILDLWVY (Nef)	4000
